# PROGNOSTIC FACTORS IN PATIENTS WITH PRESSURE SORES IN A UNIVERSITY HOSPITAL IN SOUTHERN BRAZIL

**DOI:** 10.1590/1413-785220172506169042

**Published:** 2017

**Authors:** GUSTAVO PALMEIRO WALTER, WILLIAM SEIDEL, RENATA DELLA GIUSTINA, JORGE BINS-ELY, ROSEMERI MAURICI, JANAÍNA LUZ NARCISO-SCHIAVON

**Affiliations:** 1. Plastic Surgery Division, Hospital Universitario Polydoro Ernani de Sao Thiago, Universidade Federal de Santa Catarina, Florianópolis, SC, Brazil.; 2. Postgraduate Program in Intensive and Palliative Care, Universidade Federal de Santa Catarina, Florianópolis, SC, Brazil.

**Keywords:** Pressure ulcer/epidemiology. Pressure ulcer/mortality. Surgery, plastic. Recurrence. Spinal cord injuries., Lesão por pressão/epidemiologia, Lesão por pressão/mortalidade, Cirurgia plástica, Recidiva, Traumatismos da medula espinal.

## Abstract

**Objective:**

Despite advances in medical care, patients who are hospitalized or have spinal cord injuries often develop pressure sores. The objective of this study was to describe the epidemiological characteristics of pressure sores and evaluate factors associated with recurrence and cure.

**Methods:**

In this historical cohort study, clinical and laboratory data were collected from medical records between 1997 and 2016.

**Results:**

Sixty individuals with pressure ulcers were included; mean patient age was 38.1±16.5 (37.0) years, 83.3% were men, and 86.8% identified as white. Most patients (85.1%) had paraplegia, amputation, or trauma of the lower limbs with motor sequelae; the remainder (14.9%) were quadriplegic. Most (78.3%) underwent surgery, and the mean follow-up time was 1.8±2.5 years. The lesions were cured in 25 patients; they recurred in 25% of the patients, and recurrence was seen to be associated with the location of the lesions. Patients with recurrent lesions had more medical consultations and a longer treatment time. Individuals whose ulcers had healed had fewer lesions, higher body mass index (BMI), and a higher proportion of these patients underwent surgery.

**Conclusions:**

BMI and location and number of lesions are prognostic factors. **Level of Evidence IV, Case Series.**

## RESUMO

**Objetivo:**

Apesar do progresso dos cuidados médicos, os pacientes hospitalizados ou com lesões medulares frequentemente desenvolvem úlceras de pressão. O objetivo deste estudo foi descrever as características epidemiológicas das úlceras de pressão e avaliar os fatores associados à recorrência e à cura.

**Métodos:**

Neste estudo de coorte histórico, foram coletados dados clínicos e laboratoriais de prontuários médicos de 1997 a 2016.

**Resultados:**

Sessenta indivíduos com úlceras de pressão foram incluídos. A média de idade dos pacientes foi 38,1 ± 16,5 (37,0) anos, 83,3% eram homens e 86,8% foram identificados como brancos. A maioria dos pacientes (85,1%) tinha paraplegia, amputação ou trauma nos membros inferiores com sequelas motoras; os restantes (14,9%) eram tetraplégicos. A maioria dos pacientes (78,3%) foi submetida à cirurgia e o tempo médio de acompanhamento foi 1,8±2,5 anos. A cicatrização das lesões foi observada em 25 pacientes; houve recorrência em 25% dos pacientes e verificou-se que estavam associadas à localização das lesões. Os pacientes com lesões recorrentes tinham maior número de consultas médicas e maior tempo de tratamento. Os indivíduos cujas úlceras cicatrizaram tinham menos lesões, maior índice de massa corporal (IMC) e maior proporção deles foi submetida à cirurgia.

**Conclusões:**

O IMC, a localização e o número de lesões são fatores prognósticos. **Nível de Evidência IV, Série de Casos.**

## INTRODUCTION

Pressure sores are lesions caused by local ischemia in debilitated patients, who are chronically ill or suffer from spinal cord injury. Friction, moisture and the presence of bony protuberances in contact with support surfaces are risk factors for the development of these sores.^1^

Pressure sores have a negative impact on patients’ quality of life and cause a considerable increase in hospital costs. Preventing the development of new lesions and their recurrence after treatment is fundamental to improving quality of life and reducing healthcare expenses.^2^ A study conducted in the Netherlands in 2013 found that the average cost for treatment of multiple sores was approximately 40,882 Euros.^3^ In the United States (US), 2.5 million sores are treated annually at a cost of 11 billion US dollars.^1^

Despite advancements in healthcare, the prevalence of pressure sores remains high, so much that in developing countries, more than 90% of patients with spinal cord injuries have pressure sores.^4^ The prevalence of pressure sores in hospitalized patients is 16.9% in Brazil^5^ and 18.1% in Europe.^6^ In Nebraska, the incidence is 8.5% in tertiary hospitals and 23.9% in nursing homes.^7^

Recurrence rates reach 70% after reconstructive surgery^8^ as such patients have multiple risk factors: skin insensitivity, incontinence, immobility, joint contractures, muscle spasms and other comorbidities.^2^

Few studies have identified clinical and biochemical factors related to the post-treatment prognosis of patients with pressure sores. This study aims to evaluate the clinical and biochemical characteristics of individuals with pressure sores treated at a service that is regarded as a reference in plastic surgery in South Brazil, and to identify the characteristics associated with recurrence after curative treatment.

## METHODS

### Sample

This is a cross-sectional analytical study of adult individuals with pressure sores treated at the plastic surgery outpatient clinic of Polydoro Ernani de São Thiago, a public and tertiary University Hospital of Federal University of Santa Catarina (HU/UFSC) in Florianopolis - Brazil, between 1997 and 2016. Eligible patients were identified from the outpatient and surgical attendance record, and recruited by phone calls or during routine outpatient visits. The individuals or their family members were invited to participate in the study and to sign the informed consent form. Clinical data was extracted from the medical records and biochemical data was obtained from the laboratory registration system. Patients with insufficient registration data in their medical records were excluded.

Data on the following clinical and demographic variables were collected: gender, age, race, body mass index (BMI), presence of caretaker, smoking status, alcohol status, comorbidities (hypertension, diabetes mellitus (DM), dementia, previous stroke, myelomeningocele), disability (paraplegia, injury or amputation of lower limbs, quadriplegia). The appearance of pressure sores after hospitalization, outpatient nutritional follow-up, and number of medical consultations with the plastic surgery staff were also evaluated. Severity was evaluated according to the location and the number of sores. With regard to treatment, the conduct of reparative surgery and the total time of treatment were documented. Surgical treatment was decided at the discretion of the attending plastic surgeon, and was based on clinical assessment of the patient and the severity of the lesion. 

### Laboratory tests

The following biochemical variables were analyzed: hemoglobin, leukocyte count, fasting glucose, creatinine, serum sodium and prothrombin activation time (PAT). The test results were expressed in absolute values. 

### Statistical analysis

The patients were divided into two groups: presence of recurrence, and absence of recurrence with progression to cure. Bivariate analysis was used to compare the groups with regard to each clinical and biochemical characteristic of interest.

The mean and standard deviation (SD) of normally distributed numerical variables were compared using the Student’s t-test. The medians of non-normally distributed numerical variables were compared with the Mann-Whitney test. Qualitative variables were expressed in frequencies (%); the Chi-square test or Fischer’s exact test was used to analyze them where required. Values of p<0.05 were considered to be statistically significant. 

The statistical program *Statistical Package for the Social Sciences*, version 17.0 (IBM SPSS statistics, Chicago, Illinois, US) was used to conduct the tests. 

The study protocol met the ethical precepts of the Helsinki Declaration and was approved by the Human Research Ethics Committee of UFSC under the number 1215312.

## RESULTS

### Patients characteristics

Between 1997 and 2016, 92 patients with pressure sores were identified as eligible for this study. Thirty-two patients were excluded due to incomplete clinical data.

In total, 60 patients suffering from pressure sores were included in the study. The average age was 38.1 ± 16.5 (37.0) years, 83.3% were male and 86.8% identified as white. The majority (85.1%) suffered from paraplegia, amputation or trauma of the lower limbs with motor sequelae, 14.9% suffered from quadriplegia, and 10% had myelomeningocele. 3.4% of the patients had had previous stroke, 1.7% had dementia and 1.7% had developed the sores after hospitalization.

Among those with paraplegia, 26.5% were victims of stab wounds or firearm injuries, 23.5% were victims of road accidents and 14.7% of falls. Among individuals who suffered amputation or trauma to the lower limbs, 50.0% were victims of road accidents and 50% of falls. Among those with quadriplegia, 25.0% were victims of stab wounds or firearm injuries and 25.0% of road accidents.

With regard to the location of the pressure sores, 47.5% were sacral, 37.3% ischial, 8.5% trochanteric, 3.4% calcaneal and 3,4% in other sites. Two or more sores were present in 40.7% of the patients, and the commonest combinations were sacral and trochanteric sores (33.3%), and sacral and ischial sores (29.2%).

Most (78.3%) of the patients underwent plastic surgery. The mean follow-up time was 1.8 2.5 years. Twenty-two (36.7%) were lost to follow-up, 10 (16.6%) remain under follow up, 25 (41.7%) were considered cured and 3 have (5.0%) died. Of the 60 patients, 25% have suffered from recurrent lesions at some point during follow-up. The mean time to recurrence after treatment was 0.9 2.6 years.

The clinical and biochemical characteristics of the study participants are described in Table 1.

**Table 1:** Clinical and biochemical characteristics of patients with pressure sores, according to post-treatment recurrence with the Plastic Surgery staff.

Characteristic	N	All N = 60 (100%)	Recurrence N =15 (25%)	Non-Recurrence N = 45 (75%)	P
Age*	59	38.1 ± 16.5 (37.0)	41.4 ± 17.1 (38.0)	37.0 ± 16.3 (34.5)	0.433^m^
Male, n (%)	60	50 (83.3)	11 (73.3)	39 (86.7)	0.250^f^
White, n (%)	53	46 (86.8)	11 (73.3)	35 (92.1)	0.090^f^
Body Mass Index, n (%)	17	22.2 ± 3.2 (22.0)	23.6 ± 3.7 (23.0)	21.4 ± 2.8 (21.9)	0.200^t^
Have a caretaker, n (%)	47	32 (68.1)	10(71.4)	22(66.7)	1.00^f^
Current Smoker, n (%)	51	12 (23.5)	3(23.1)	9(23.7)	1.00^f^
Current Drinker, n (%)	49	2 (4.1)	1(7.7)	1(2.8)	0.464^f^
Diabetes Mellitus, n (%)	60	2(3.3)	0	2(4.4)	1.00^f^
Hypertension, n (%)	60	3(5)	2(3.3)	1(2.2)	0.151^f^
Stroke, n (%)	58	2(3.4)	1(7.1)	1(2.3)	0.428^f^
Dementia, n (%)	59	1(1.7)	0	1(2.2)	1.000^f^
Myelomeningocele, n (%)	60	6(10)	2(13.3)	4(8.9)	0.634^f^
Disability:	47				0.086^f^
--- Paraplegia/Limb amputation, n (%)		40 (85.1)	14 (100)	26 (78.8)	
--- Quadriplegia, n (%)		7 (14.9)	0 (0.0)	7 (21.2)	
Lesion after Hospitalization, n (%)	60	1 (1.7)	0	1 (2.2)	1.00^f^
Outpatient Nutritional Follow-up, n (%)	60	7 (11.7)	2 (3.3)	5 (11.1)	1.00^f^
Over 8 visits to plastic surgery, n (%)	60	29 (48.3)	12 (80)	17 (37.8)	0.005^q^
Two or more sores, n (%)	59	24 (40.7)	8 (57.1)	16 (35.6)	0.151^q^
Ulcer location:	59				0.037^q^
--- Sacral, n (%)		28 (47.5)	8 (57.1)	20 (44.4)	
--- Ischial, n (%)		22 (37.3)	2 (14.3)	20 (44.4)	
--- Trochanteric, n (%)		5 (8.5)	1 (7.1)	4 (8.1)	
--- Calcaneus, n (%)		2 (3.4)	1 (7.1)	1 (2.2)	
--- Others		2 (3.4)	2 (14.3)	0 (0.0)	
Surgery, n (%)	60	47 (78.3)	14 (93.3)	33 (73.3)	0.153^f^
Cure, n (%)	38	25 (65.8)	8 (72.7)	17(63)	0.714^f^
Treatment time (year)*	54	1.8 ± 2.5 (1.0)	3.7 ± 3.5(1.8)	1.2 ± 1.6 (0.8)	0.007^m^
Death, n (%)	38	3 (7.9)	1 (9.1)	2 (7.4)	1.00^f^
Hemoglobin* (g/dl)	45	11.4 ± 3.0 (12.2)	11.4 ± 3.4 (12.1)	11.4 ± 2.9 (12.2)	0.943^t^
Leukocytes* (thousand/mm^3^)	42	8967 ± 3622 (7950)	9250 ± 4202 (8250)	8854 ± 3436 (7880)	0.759^m^
Fasting glucose* (g/dl)	27	93.8 ± 18.3 (91)	96.7 ± 26.0 (91.0)	93.0 ± 16.3 (89.0)	0.953^m^
Creatinine* (mg/dl)	34	0.8 ± 0.3 (0.7)	0.8 ± 0.3 (0.8)	0.7 ± 0.3 (0.7)	0.236^m^
Sodium* (mEq/L)	23	138.1 ± 4.4 (139.0)	138.3 ± 2.4 (139.0)	138.1 ± 25.1 (138.5)	0.759^m^
Prothrombin activity time*	24	78.0 ± 12.0 (76.8)	82.3 ± 13.3 (77.5)	75.8 ± 11.1 (76.8)	0.216^t^

*Mean ± standard deviation (median); m: Mann Whitney test; f: Fisher’s exact test; q: chi-square test; t: Student’s t-test.

### Clinical and biochemical characteristics of individuals with pressure sores, according to recurrence of lesions after treatment

On comparing individuals with and without recurrence after treatment, (Table 1) recurrence was found to be associated with a higher rate (8 or more) of outpatient visits with the plastic surgery team (80% *vs.* 37.8%; P = 0.005) and longer follow-up (1.8 vs*.* 0.8 years; P = 0.007). There was a difference in recurrence rate according to the location of the sores (P = 0.037): a higher rate of sores recurred in the sacral region (57.1 vs*.* 44.4%) and a lower rate in the ischial region (14.3 vs*.* 44.4%). There was no difference in relation to age, gender, race, BMI, presence of caretaker, smoking status, alcohol status, comorbidities (SAH, DM, CVA, myelomeningocele), disabilities (paraplegia, injury or amputation of lower limbs, quadriplegia, appearance of lesion during hospitalization, outpatient nutritional follow-up, number of sores, conduct of reparative surgery, hemoglobin levels, leukocytes, fasting glucose levels, creatinine, sodium and PAT.

### Clinical and biochemical characteristics of individuals suffering from pressure sores, according to the healing of the lesions

Individuals who were completely healed had a higher median BMI (23.3 *vs.* 19.7 kg/m^2^; P = 0.024), higher mean hemoglobin (12.2 2.5 *vs.* 9.7 3.6 g/dl; P = 0.033) and higher rate of undergoing plastic surgery (92.0% *vs.* 61.5%; P = 0.034). (Table 2) This group had the lowest proportion of individuals with two or more pressure sores in various regions (28.0% vs. 66.7%; P = 0.036). There was no difference in relation to age, gender, race, presence of caretaker, smoking status, alcohol status, comorbidities (SAH, DM, CVA, myelomeningocele), disabilities (paraplegia, injury or amputation of lower limbs, quadriplegia, prolonged hospitalization, outpatient nutritional follow-up, number of visits with plastic surgery team, leukocyte count, fasting glucose levels, creatinine, sodium and PAT.

**Table 2:** Clinical and biochemical characteristics associated with cure after plastic surgery treatment.

Characteristic	N	All n = 38 (100%)	Cure N = 25 (66%)	Non-Cure N = 13 (34%)	P
Age*	37	39.2 ± 17.4 (35.0)	35.4 ± 14.5 (33.0)	46.2 +- 20.4 (43)	0.071^t^
Male, n (%)	38	30 (78.9)	21 (84.0)	9 (69.2)	0.407^f^
White skin color, n (%)	33	29 (87.9)	19 (86.4)	10 (90.9)	1.000^f^
Body mass index*	14	22.5 ± 3.4 (22.1)	23.6 ± 3.3 (23.3)	19.8 ± ± 1.8 (19.7)	0.024^m^
Have a caretaker, n (%)	32	23 (71.9)	16 (76.2)	7 (63.6)	0.681^f^
Current Smoking, n (%)	34	4 (11.8)	2 (8.3)	2 (20)	0.564^f^
Current Alcoholism, n (%)	33	1 (3.0)	0 (0.0)	1 (11.1)	0.273^f^
Diabetes mellitus, n (%)	38	2 (5.3)	0 (0.0)	2 (15.4)	0.111^f^
Hypertension, n (%)	38	3 (7.9)	1 (4.0)	2 (15.4)	0.265^f^
Stroke, n (%)	37	2 (5.4)	0 (0.0)	2 (15.4)	0.117^f^
Dementia, n (%)	37	1 (2.7)	0 (0.0)	1 (7.7)	0.351^f^
Myelomeningocele, n (%)	38	5 (13.2)	3 (12.0)	2 (15.4)	1.000^f^
Disability:	32				0.637^f^
---Paraplegia or Limb Amputation, n (%)		26 (81.3)	17 (77.3)	9 (90)	
--- Quadriplegia, n (%)		6 (18.8)	5 (22.7)	1(10.0)	
Lesion after hospitalization, n (%)	38	1 (2.6)	0 (0.0)	1 (7.7)	0.342^f^
Outpatient nutritional follow-up, n (%)	38	5 (13.2)	4 (16.0)	1 (7.7)	0.643^f^
Eight or more visits, n (%)	38	20 (52.6)	14 (56.0)	6 (46.2)	0.734^f^
Two or more sores, n (%)	37	15 (40.5)	7 (28.0)	8 (66.7)	0.036^f^
Surgery, n (%)	38	31 (81.6)	23(92.0)	8 (61.5)	0.034^f^
Death, n (%)	38	3 (7.9)	1(9.1)	2 (7.4)	1.000^f^
Hemoglobin (g/dl) *	28	11.4 ± 3.0 (12.2)	12.2 ± 2.5 (12.9)	9.7 ± 3.6 (10.5)	0.033^t^
Leukocytes (/mm^3^) *	26	9.083 ± 4.168 (8.000)	8.411 ± 3.773 (7.144)	10.353 ± 4.800 (9.930)	0.178^m^
Fasting glucose (g/dl) *	14	91.7 ± 19.5 (90.0)	88.6 ± 12.7 (89.0)	97.8 ± 29.0 (92.0)	0.417^t^
Creatinine (mg/dl) *	18	0.8 ± 0.3 (0.8)	0.7 ± 0.3 (0.7)	0.9 ± 0.3 (0.8)	0.112^m^
Sodium (mEq/L) *	15	138.8 ± 4.8 ± (139.0)	137.9 ± 2.4 (139)	139.7 ± 6.7 (138)	0.857^m^
Prothrombin activity time *	21	81.5 ± 11.0 (78.5)	81.3 ± 12.1 (76.8)	81.9 ± 9.7 (80.4)	0.945^t^

*Mean ± standard deviation (median); t: Student’s t-test; f: Fisher’s exact test; m: Mann Whitney’s test.

## DISCUSSION

The mean age of the participants in our study is similar to the 29 to 34 years described by Arora et al.^9^ and Costa et al.^10^ These differ from those of other studies, which had a mean participant age ranging between 56 to 60 years. This is because their study population comprised patients with lesions secondary to immobility from long periods of hospitalization.^11^ ^-^ ^14^ In our study, only a minority of patients (7%) fitted this profile. The majority of our patients had spinal or congenital (myelomeningocele) traumatic lesions similar to those described by Yamamoto et al.,^13^ in which 49% of the paraplegic patients had experienced trauma. Pressure sores are commoner in males,^10^ ^,^ ^13^ ^,^ ^14^ possibly because they tend to be more exposed to situations involving the risk of trauma with spinal cord injury.^10^

With regard to the location of pressure sores, sacral sores tend to be commonest, varying from 72 to 87% in Hospital São Paulo^14^ ^-^ ^16^ and 32% in Hospital das Clínicas.^10^ These findings are similar to ours, and suggests that many of these patients remain in the dorsal decubitus position for a prolonged time.

The treatment of pressure sores can be divided into systemic and local options, and the latter can be subdivided into conservative and surgical. Surgical treatment of pressure sores is the therapeutic option of last resort, and is indicated for wounds refractory to clinical treatment or when fast scarring of the lesion is required.^17^ Even then, reparative plastic surgery is indicated in 71% to 78% of patients.^2^ ^,^ ^10^ The recurrence rate of lesions is usually high and ranges from 11% to 63%.^10^ ^,^ ^11^ ^,^ ^13^ ^,^ ^18^ ^-^ ^20^ Recurrence rates in the last century reached 70%.^8^ The high recurrence rate even after treatment implies that the initial causative factors had not been resolved, and also that complications persist.^11^ In United Kingdom, low recurrence rates (6% after 33 months) are due to the implementation of a multidisciplinary patient follow-up program not reported in the other studies.^2^

With regard to prognostic factors, smoking and comorbidities have been associated with an increased prevalence of pressure sores in patients with spinal cord injury.^21^ Similarly, Berlowitz et al.^22^ demonstrated that bedridden or wheelchair-bound patients with low hemoglobin levels have a lower rate of cure of pressure sores. Later, the same group identified factors significantly associated with the presence of sores, including change in level of awarenes, being bedridden or wheelchair bound, poor nutritional intake and hypoalbuminemia.^23^ However, there is little evidence to justify the routine use of nutritional supplements, biological agents and adjuvant therapies when compared with standard therapies.^24^

Recurrence rates are the main problem in pressure sore reconstructions. Recurrence has been associated to glycated hemoglobin level exceeding 6%, repeating the same flap already used in a surgery that recurred^18^ and being African-American.^11^ ^,^ ^25^ Skin color did not attain statistical significance in our study, and Guihan et al.^11^ notes a probable *bias* in their race criterion related to the socioeconomic status of the patients evaluated. The permanence of cure does not depend on adequate surgical treatment in selected patients, but rather on a combination of factors. Multidisciplinary care, focus on the nutritional state of the patient, and use of prophylactic measures are important.^1^

We would like to address some limitations to the present study. Considering the prevalence of pressure sores in the general population, the number of patients included in the study is small. However, the University Hospital is a tertiary center for plastic surgery and cares for individuals from the entire state of Santa Catarina, which is one of the smallest states in Brazil. In addition, the study’s sample size is similar to that of other studies on pressure sores.^2^ ^,^ ^10^ ^,^ ^11^ ^,^ ^13^ ^,^ ^20^ ^,^ ^22^ One of the problems in understanding the recurrence of pressure sores is the lack of clear terminology for evaluating sores that develop in the same anatomical region. When a sore develops, it may represent an incomplete healing of the previously treated sore or a new lesion adjacent to a healed sore. In our study, we defined recurrence as the appearance of a lesion in a previously treated location that had been considered healed after clinical assessment by the attending physician. As this is a retrospective study, specific characteristics relating to the severity of the ulcers (e.g. size and depth) were not available from the medical records and thus could not be described. Finally, the study could not evaluate the surgical techniques used to treat the pressure sores, as these depended on the discretion of the attending physician and the patient’s clinical state.

This study enables us to conclude that the commonest cause of pressure sores are spinal cord injuries associated with trauma or congenital diseases, and they are most commonly located in the sacral and ischial regions. The majority of these patients undergo plastic surgery, and the recurrence rates of post-treatment lesions are similar to those found worldwide. Recurrence is associated with the location of the lesions, higher number of medical consultations and longer time of treatment. Cure is associated with higher BMI, higher mean hemoglobin, lower number of sores and plastic surgery treatment.
